# Crystal structure of [4-(chloro­meth­yl)phen­yl](4-hy­droxy­piperidin-1-yl)methanone

**DOI:** 10.1107/S2056989015016096

**Published:** 2015-09-12

**Authors:** B. K. Revathi, D. Reuben Jonathan, K. Kalai Sevi, K. Dhanalakshmi, G. Usha

**Affiliations:** a PG and Research Department of Physics, Queen Mary’s College, Chennai-4, Tamilnadu, India; b Department of Chemistry, Madras Christian College, Chennai-59, India; c SCRI, Anna hospital Campus, Chennai-106, Tamilnadu, India; d Anna Siddha Medical College, Chennai-106, Tamilnadu, India

**Keywords:** crystal structure, piperidine derivative, hydrogen bonding

## Abstract

The title compound, C_13_H_16_ClNO_2_, crystallized with two independent mol­ecules in the asymmetric unit (*A* and *B*). The piperidinol ring in mol­ecule *B* is disordered over two positions with a site occupancy ratio of 0.667 (5):0.333 (5). In both mol­ecules these rings have a chair conformation, including the minor component in mol­ecule *B*. Their mean planes are inclined to the benzene ring by 45.57 (13)° in mol­ecule *A*, and by 50.5 (4)° for the major component of the piperidine ring in mol­ecule *B*. In the crystal, the individual mol­ecules are linked by O—H⋯O hydrogen bonds, forming chains of *A* and *B* mol­ecules along the [100] direction. The chains are inter­linked by C—H⋯O hydrogen bonds, forming ribbons.

## Related literature   

For the synthesis see: Revathi *et al.* (2015 ▸). For the biological activity of piperidine derivatives, see: Daly *et al.* (1986 ▸); Fodor *et al.* (1985 ▸); Campfield *et al.* (1995 ▸); Kozikowski *et al.* (1998 ▸); Brau *et al.* (2000 ▸); Bolzani *et al.* (1995 ▸); Gulluoglu *et al.* (2007 ▸). For related structures see: Revathi *et al.* (2015 ▸); Prathebha *et al.* (2015 ▸).
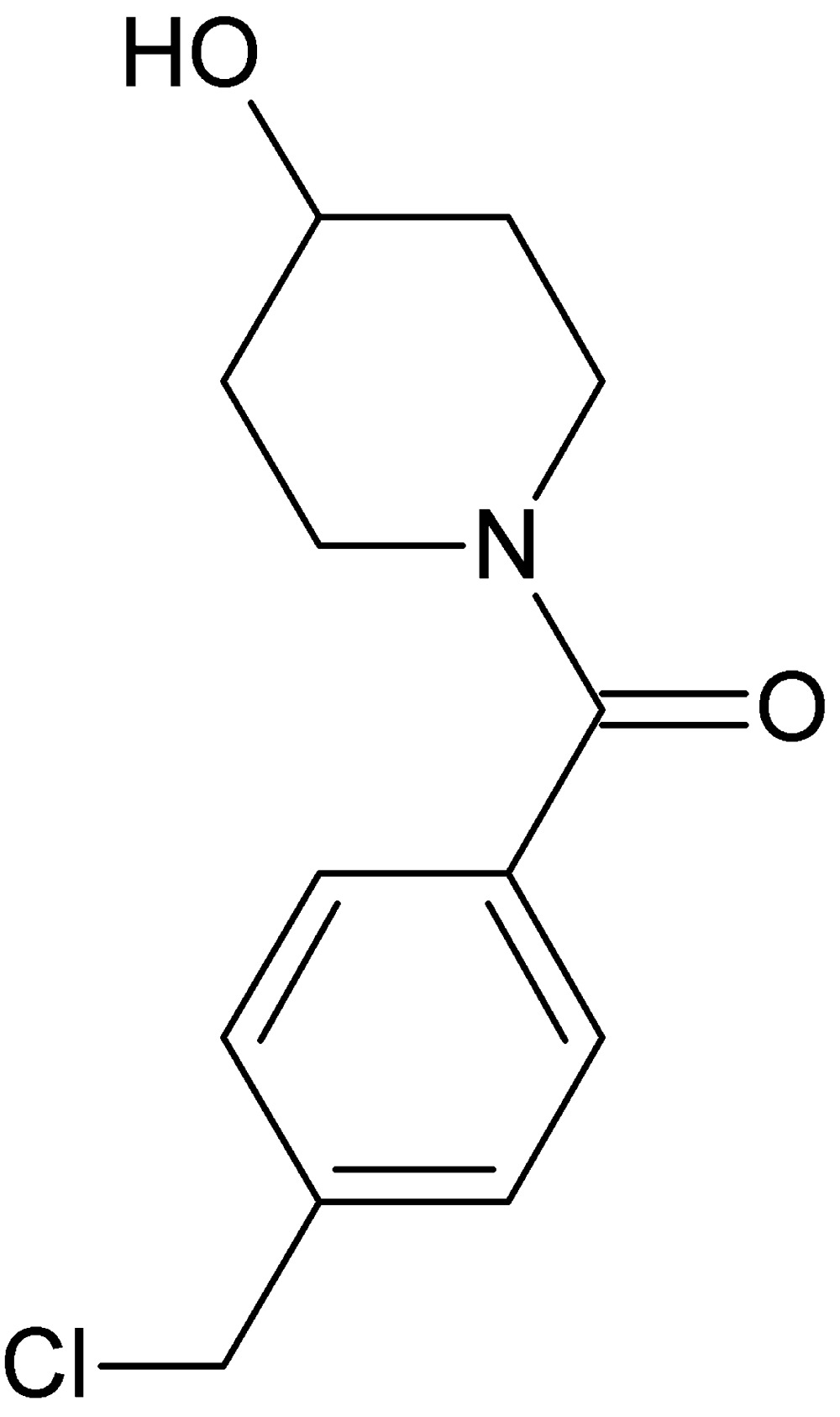


## Experimental   

### Crystal data  

C_13_H_16_ClNO_2_
*M* *_r_* = 253.72
Triclinic, 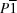

*a* = 8.4131 (3) Å
*b* = 9.6211 (4) Å
*c* = 15.6780 (6) Å
α = 80.917 (3)°
β = 89.240 (2)°
γ = 88.395 (2)°
*V* = 1252.57 (8) Å^3^
*Z* = 4
Mo *K*α radiation
μ = 0.29 mm^−1^
*T* = 293 K
0.35 × 0.30 × 0.25 mm

### Data collection  

Bruker Kappa APEXII CCD diffractometer
Absorption correction: multi-scan (*SADABS*; Bruker, 2004 ▸) *T*_min_ = 0.902, *T*_max_ = 0.943
28447 measured reflections
4415 independent reflections
3411 reflections with *I* > 2σ(*I*)
*R*_int_ = 0.027

### Refinement  

*R*[*F* ^2^ > 2σ(*F* ^2^)] = 0.045
*wR*(*F* ^2^) = 0.128
*S* = 1.05
4415 reflections
371 parameters
111 restraints
H-atom parameters constrained
Δρ_max_ = 0.45 e Å^−3^
Δρ_min_ = −0.62 e Å^−3^

### 

Data collection: *APEX2* (Bruker, 2004 ▸); cell refinement: *APEX2* and *SAINT* (Bruker, 2004 ▸); data reduction: *SAINT* and *XPREP* (Bruker, 2004 ▸); program(s) used to solve structure: *SIR92* (Altomare *et al.*, 1993 ▸); program(s) used to refine structure: *SHELXL2014* (Sheldrick, 2015 ▸); molecular graphics: *ORTEP-3 for Windows* (Farrugia, 2012 ▸) and *Mercury* (Bruno *et al.*, 2002 ▸); software used to prepare material for publication: *SHELXL2014*.

## Supplementary Material

Crystal structure: contains datablock(s) I, New_Global_Publ_Block. DOI: 10.1107/S2056989015016096/bg2567sup1.cif

Structure factors: contains datablock(s) I. DOI: 10.1107/S2056989015016096/bg2567Isup2.hkl

Supporting information file. DOI: 10.1107/S2056989015016096/bg2567Isup3.cml

**Fig. 1.**
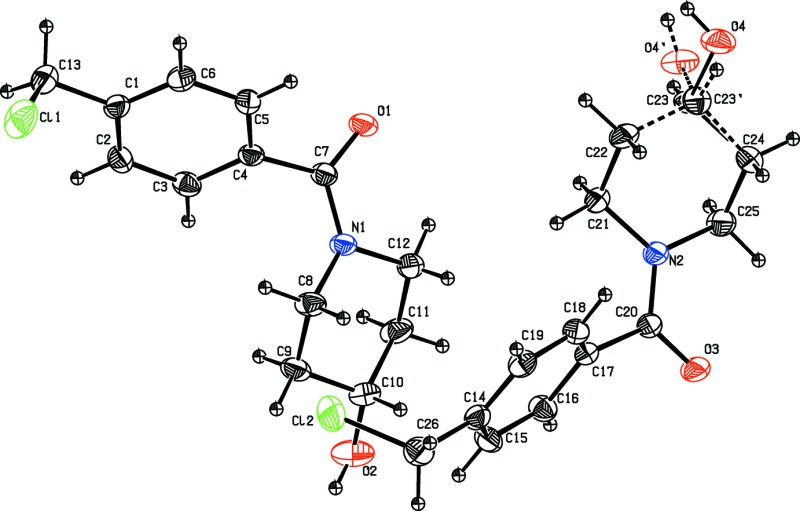
The molecular structure of the title compound (left: molecula A; right: molecule B), with displacement ellipsoids drawn at the 30% probability level.

. DOI: 10.1107/S2056989015016096/bg2567fig1.tif
The mol­ecular structure of the title compound (left: mol­ecula A; right: mol­ecule B), with displacement ellipsoids drawn at the 30% probability level.

**Fig. 2.**
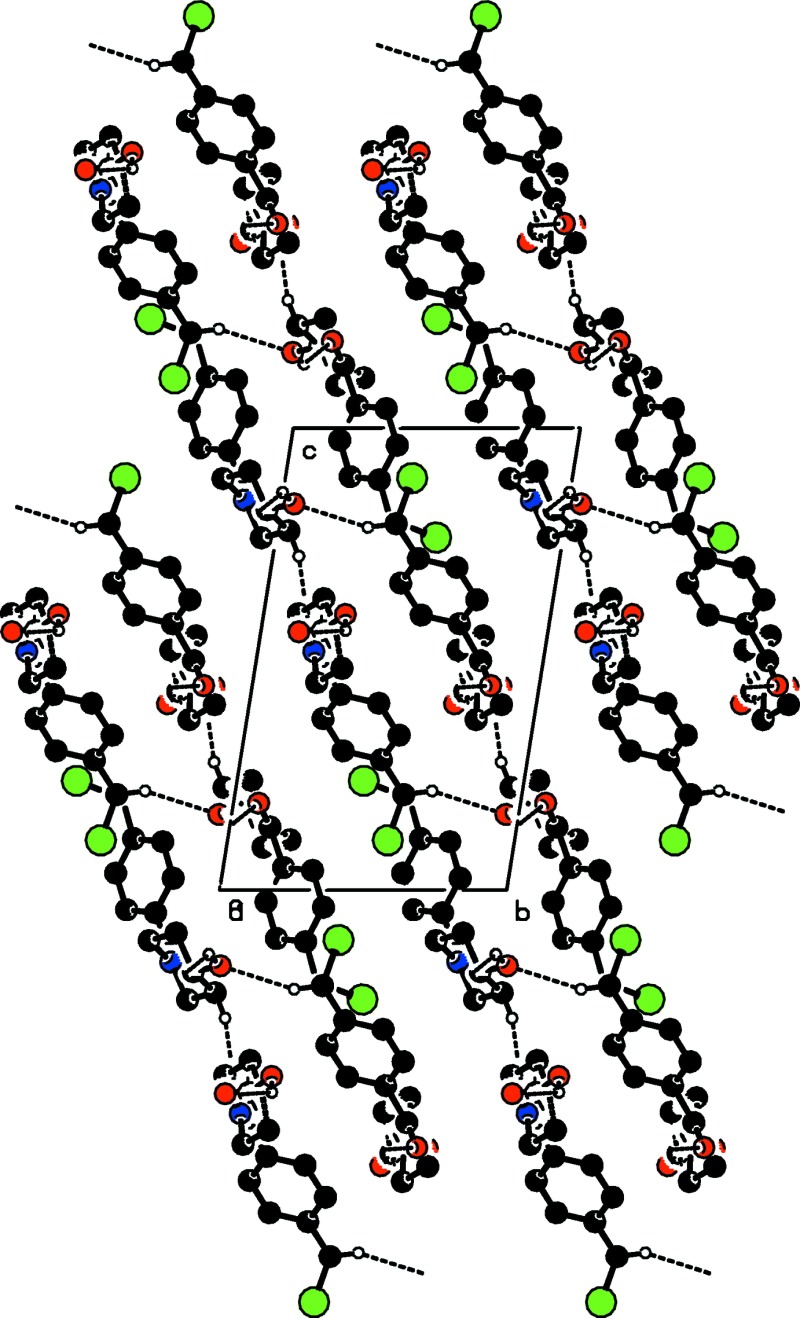
The packing of the molecules in the crystal structure. The dashed lines indicate the hydrogen bonds.

. DOI: 10.1107/S2056989015016096/bg2567fig2.tif
The packing of the mol­ecules in the crystal structure. The dashed lines indicate the hydrogen bonds.

CCDC reference: 1421009

Additional supporting information:  crystallographic information; 3D view; checkCIF report

## Figures and Tables

**Table 1:** Hydrogen-bond geometry (, )

*D*H*A*	*D*H	H*A*	*D* *A*	*D*H*A*
O2H2*A*O1^i^	0.82	2.18	2.789(2)	132
O4H4O3^ii^	0.82	2.13	2.793(3)	138
C11H11*B*O3^iii^	0.97	2.60	3.522(3)	160
C26H26*A*O2^iv^	0.97	2.59	3.494(4)	154

Symmetry codes: (i) 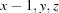; (ii) 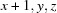; (iii) 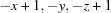; (iv) 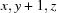.

## supplementary crystallographic information

### S1. Comment

Many piperidine containing compounds possess remarkable biological and medicinal properties (Daly *et al.*, (1986); Fodor *et al.*, (1985)). Among their remarkable properties, they show appreciable effect on plasma glucose level (Campfield *et al.*, (1995)), insulin normalization, therapeutics on cocaine abuse (Kozikowski *et al.*, (1998)). Piperidine also participates in amny local anesthetics, such as mepivacaine, ropivacaine, and bupivacaine, extensively used in clinical practice (Brau *et al.*, (2000); Bolzani *et al.*, (1995)). Piperidine derivatives are found to exhibit pharmacological activity and form a vital part of the molecular structures of important drugs such as raloxifene and minoxidil. Selective inhibition of a number of enzymes has rendered piperidine alkaloids as important paraphernalia in the study of biochemical pathways (Gulluoglu *et al.*, (2007).

The title compound, C_13_H_16_ClNO_2_, (I), crystallizes with two molecules in the asymmetric unit: A ( Fig1, left) and B (Fig 1, right) . Bond lengths and angles are comparable with literature values. C—N distances of the piperdine ring in molecule A C8—C12/N1 & in molecule B C21—C25/N2, are in the range 1.459 (3)- 1.462 (3) Å and are in good agreement with values of a similar reported structure (Revathi *et al.*, (2015)). The C=O distances in molecules A & B are [1.235 (3) and 1.233 (3) Å], respectively, and is comparable with the previously reported value(Prathebha *et al.*, (2015). In the molecule A, the dihedral angle between piperdine ring and the phenyl ring 47.22 (1)°, indicates the bisectional orientation of the phenylring. The bond angles around the N1 and N2 atoms [358.85 (2)° and 359.47 (2)°, respectively], shows *sp*^2^ hybridization of the atoms. The piperidine ring of the molecule A, adopts a chair conformation with puckering parameters of q2 = 0.019 (2) Å, φ2 = -58.74° q3 = -0.567 (3) Å, QT= 0.567 (3) Å and θ2 = 178.03 (2)°.

In the crystal packing the molecules form chains running along the diagonal of '*bc*' plane through O—H···O type hydrogen bonds. These chains are further inter linked through C—H···O type hydrogen bonds to form molecular ribbons (Fig. 2).

### S2. Experimental

The title compound was synthesized following a publish procedure (Revathi *et al.*, (2015)). In a 250 ml roundbottomed flask 120 ml of ethylmethylketone was added to 4-hydroxypiperdine (0.02 mol) and stirred at room temperature. After 5 min triethylamine (0.04 mol) was added and the mixture was stirred for 15 min. Then 4-chloromethyl benzoylchloride(0.04 mol) was added and the reaction mixture was stirred at room temperature for *ca* 2 h. A white precipitate of triethylammoniumchloride was formed. It was filtered and the filtrate was evaporated to give the crude product. It was recrystallized twice from ethylmethylketone (yield: 82%) giving colourless block-like crystals of the title compound.

### S3. Refinement

H atoms were positioned geometrically and treated as riding on their parent atoms and refined with, C—H distance of 0.93–0.98 Å, O—H distance of 0.82 Å with *U*_iso_(H)= 1.5 *U*_eq_(c-methyl),*U*_iso_(H)= 1.5 *U*_eq_(O) and *U*_iso_(H)= 1.2Ueq(C) for other H atom. The piperidinol ring in one of the molecules is disordered over two positions with site occupancies in the ratio 67:33. The disorder was resolved by successive Fourier electron density maps and least squares refinements. Sum of the occupancies of the disordered components were restrained as 1 during refinement. The bond distances in the disordered groups were restrained using SADI or *DFIX* with an effective standard deviation of 0.01 Å and 0.02 Å respectively, wherever necessary. Rigid group restraint(RIGU) with e.s.d.'s 0.002 Å and 0.004 Å was also applied to get satisfactory model of the disorder.

### Crystal data

**Table:**

C_13_H_16_ClNO_2_	*Z* = 4
*M**_r_* = 253.72	*F*(000) = 536
Triclinic, *P*1	*D*_x_ = 1.345 Mg m^−^^3^
*a* = 8.4131 (3) Å	Mo *K*α radiation, λ = 0.71073 Å
*b* = 9.6211 (4) Å	Cell parameters from 9919 reflections
*c* = 15.6780 (6) Å	θ = 2.3–28.1°
α = 80.917 (3)°	µ = 0.29 mm^−^^1^
β = 89.240 (2)°	*T* = 293 K
γ = 88.395 (2)°	Block, colourless
*V* = 1252.57 (8) Å^3^	0.35 × 0.30 × 0.25 mm

### Data collection

**Table:**

Bruker Kappa APEXII CCD diffractometer	4415 independent reflections
Radiation source: fine-focus sealed tube	3411 reflections with *I* > 2σ(*I*)
Graphite monochromator	*R*_int_ = 0.027
ω and φ scan	θ_max_ = 25.0°, θ_min_ = 1.3°
Absorption correction: multi-scan (*SADABS*; Bruker, 2004)	*h* = −10→10
*T*_min_ = 0.902, *T*_max_ = 0.943	*k* = −11→11
28447 measured reflections	*l* = −18→18

### Refinement

**Table:**

Refinement on *F*^2^	111 restraints
Least-squares matrix: full	Hydrogen site location: inferred from neighbouring sites
*R*[*F*^2^ > 2σ(*F*^2^)] = 0.045	H-atom parameters constrained
*wR*(*F*^2^) = 0.128	*w* = 1/[σ^2^(*F*_o_^2^) + (0.0477*P*)^2^ + 1.0906*P*] where *P* = (*F*_o_^2^ + 2*F*_c_^2^)/3
*S* = 1.05	(Δ/σ)_max_ = 0.001
4415 reflections	Δρ_max_ = 0.45 e Å^−^^3^
371 parameters	Δρ_min_ = −0.61 e Å^−^^3^

### Special details

**Table:**

Geometry. All e.s.d.'s (except the e.s.d. in the dihedral angle between two l.s. planes) are estimated using the full covariance matrix. The cell e.s.d.'s are taken into account individually in the estimation of e.s.d.'s in distances, angles and torsion angles; correlations between e.s.d.'s in cell parameters are only used when they are defined by crystal symmetry. An approximate (isotropic) treatment of cell e.s.d.'s is used for estimating e.s.d.'s involving l.s. planes.

### Fractional atomic coordinates and isotropic or equivalent isotropic displacement parameters (Å^2^)

**Table:**

	*x*	*y*	*z*	*U*_iso_*/*U*_eq_	Occ. (<1)
C1	1.1123 (3)	0.3281 (3)	−0.11147 (15)	0.0386 (5)	
C2	1.0000 (3)	0.2251 (3)	−0.10479 (15)	0.0413 (6)	
H2	0.9657	0.1937	−0.1544	0.050*	
C3	0.9381 (3)	0.1683 (3)	−0.02565 (16)	0.0408 (6)	
H3	0.8600	0.1011	−0.0223	0.049*	
C4	0.9916 (2)	0.2108 (2)	0.04902 (15)	0.0347 (5)	
C5	1.1051 (3)	0.3129 (3)	0.04244 (16)	0.0409 (6)	
H5	1.1425	0.3421	0.0921	0.049*	
C6	1.1633 (3)	0.3716 (3)	−0.03693 (16)	0.0449 (6)	
H6	1.2381	0.4416	−0.0405	0.054*	
C7	0.9414 (3)	0.1401 (2)	0.13670 (15)	0.0369 (5)	
C8	0.6599 (3)	0.2173 (3)	0.10907 (17)	0.0449 (6)	
H8A	0.7011	0.2648	0.0544	0.054*	
H8B	0.6232	0.2885	0.1427	0.054*	
C9	0.5227 (3)	0.1276 (3)	0.09289 (17)	0.0480 (6)	
H9A	0.5571	0.0624	0.0546	0.058*	
H9B	0.4377	0.1872	0.0647	0.058*	
C10	0.4611 (3)	0.0460 (3)	0.17647 (17)	0.0448 (6)	
H10	0.4214	0.1130	0.2133	0.054*	
C11	0.5957 (3)	−0.0414 (3)	0.22218 (19)	0.0545 (7)	
H11A	0.6322	−0.1113	0.1876	0.065*	
H11B	0.5578	−0.0904	0.2772	0.065*	
C12	0.7328 (3)	0.0503 (3)	0.23722 (16)	0.0538 (7)	
H12A	0.6993	0.1144	0.2763	0.065*	
H12B	0.8201	−0.0084	0.2637	0.065*	
C13	1.1795 (3)	0.3886 (3)	−0.19750 (17)	0.0512 (7)	
H13A	1.1600	0.3256	−0.2385	0.061*	
H13B	1.2937	0.3963	−0.1928	0.061*	
C14	0.3857 (3)	0.4916 (3)	0.28225 (15)	0.0401 (6)	
C15	0.3397 (3)	0.3530 (3)	0.30142 (16)	0.0449 (6)	
H15	0.2663	0.3191	0.2665	0.054*	
C16	0.4011 (3)	0.2651 (3)	0.37143 (16)	0.0423 (6)	
H16	0.3673	0.1728	0.3841	0.051*	
C17	0.5127 (3)	0.3125 (2)	0.42329 (14)	0.0348 (5)	
C18	0.5592 (3)	0.4505 (3)	0.40463 (15)	0.0412 (6)	
H18	0.6343	0.4836	0.4390	0.049*	
C19	0.4949 (3)	0.5397 (3)	0.33533 (16)	0.0418 (6)	
H19	0.5252	0.6330	0.3242	0.050*	
C20	0.5672 (3)	0.2185 (3)	0.50338 (15)	0.0376 (5)	
C26	0.3178 (3)	0.5885 (3)	0.20677 (17)	0.0529 (7)	
H26A	0.3322	0.6854	0.2146	0.063*	
H26B	0.2045	0.5738	0.2036	0.063*	
O1	1.04414 (19)	0.0877 (2)	0.18842 (11)	0.0515 (5)	
N1	0.7862 (2)	0.1306 (2)	0.15550 (12)	0.0401 (5)	
O2	0.3366 (2)	−0.0447 (2)	0.16381 (16)	0.0681 (6)	
H2A	0.2626	0.0017	0.1392	0.102*	
O3	0.4671 (2)	0.1695 (2)	0.55717 (12)	0.0556 (5)	
Cl1	1.09363 (11)	0.55854 (9)	−0.23667 (5)	0.0747 (3)	
Cl2	0.41251 (10)	0.55824 (10)	0.10800 (5)	0.0706 (3)	
C21	0.8443 (13)	0.2288 (15)	0.4478 (7)	0.0369 (19)	0.667 (5)
H21A	0.8004	0.2904	0.3983	0.044*	0.667 (5)
H21B	0.8764	0.1400	0.4299	0.044*	0.667 (5)
C22	0.9860 (15)	0.2956 (12)	0.4818 (9)	0.044 (2)	0.667 (5)
H22A	1.0703	0.3027	0.4386	0.053*	0.667 (5)
H22B	0.9558	0.3904	0.4907	0.053*	0.667 (5)
C23	1.0491 (9)	0.2147 (8)	0.5655 (5)	0.0479 (19)	0.667 (5)
H23	1.0836	0.1191	0.5582	0.057*	0.667 (5)
C24	0.9138 (13)	0.2112 (15)	0.6311 (9)	0.049 (2)	0.667 (5)
H24A	0.9507	0.1668	0.6875	0.059*	0.667 (5)
H24B	0.8774	0.3064	0.6354	0.059*	0.667 (5)
C25	0.7790 (15)	0.1296 (10)	0.6030 (5)	0.043 (2)	0.667 (5)
H25A	0.8151	0.0347	0.5977	0.052*	0.667 (5)
H25B	0.6932	0.1240	0.6452	0.052*	0.667 (5)
O4	1.1723 (3)	0.2855 (3)	0.5966 (2)	0.0623 (11)	0.667 (5)
H4	1.2493	0.2861	0.5639	0.093*	0.667 (5)
N2	0.7232 (8)	0.2047 (16)	0.5184 (5)	0.034 (2)	0.667 (5)
C21'	0.850 (3)	0.210 (3)	0.4530 (15)	0.042 (5)	0.333 (5)
H21C	0.8069	0.2460	0.3966	0.051*	0.333 (5)
H21D	0.9095	0.1236	0.4483	0.051*	0.333 (5)
C22'	0.967 (3)	0.318 (3)	0.4775 (18)	0.040 (4)	0.333 (5)
H22C	1.0482	0.3387	0.4333	0.048*	0.333 (5)
H22D	0.9105	0.4054	0.4843	0.048*	0.333 (5)
C23'	1.0410 (17)	0.2493 (16)	0.5636 (10)	0.044 (3)	0.333 (5)
H23'	1.1000	0.3196	0.5883	0.053*	0.333 (5)
C24'	0.915 (3)	0.184 (3)	0.6302 (19)	0.044 (4)	0.333 (5)
H24C	0.9685	0.1185	0.6751	0.053*	0.333 (5)
H24D	0.8646	0.2576	0.6571	0.053*	0.333 (5)
C25'	0.787 (3)	0.105 (2)	0.5893 (10)	0.036 (3)	0.333 (5)
H25C	0.8326	0.0158	0.5781	0.043*	0.333 (5)
H25D	0.7011	0.0843	0.6307	0.043*	0.333 (5)
O4'	1.1380 (7)	0.1335 (6)	0.5593 (4)	0.0532 (19)	0.333 (5)
H4'	1.1397	0.1163	0.5097	0.080*	0.333 (5)
N2'	0.7230 (17)	0.178 (3)	0.5116 (12)	0.034 (4)	0.333 (5)

### Atomic displacement parameters (Å^2^)

**Table:**

	*U*^11^	*U*^22^	*U*^33^	*U*^12^	*U*^13^	*U*^23^
C1	0.0315 (12)	0.0437 (14)	0.0382 (13)	0.0044 (10)	0.0024 (10)	0.0000 (10)
C2	0.0428 (14)	0.0448 (14)	0.0359 (13)	0.0018 (11)	−0.0064 (10)	−0.0049 (11)
C3	0.0367 (13)	0.0384 (13)	0.0454 (14)	−0.0061 (10)	−0.0063 (10)	0.0013 (11)
C4	0.0264 (11)	0.0372 (12)	0.0381 (12)	0.0032 (9)	−0.0023 (9)	0.0006 (10)
C5	0.0358 (13)	0.0497 (15)	0.0375 (13)	−0.0078 (11)	−0.0008 (10)	−0.0066 (11)
C6	0.0377 (13)	0.0499 (15)	0.0468 (14)	−0.0137 (11)	0.0038 (11)	−0.0047 (12)
C7	0.0300 (12)	0.0394 (13)	0.0391 (13)	−0.0008 (10)	−0.0044 (10)	0.0003 (10)
C8	0.0323 (13)	0.0430 (14)	0.0540 (15)	0.0056 (10)	−0.0027 (11)	0.0084 (12)
C9	0.0319 (13)	0.0584 (17)	0.0506 (15)	0.0033 (11)	−0.0103 (11)	0.0008 (12)
C10	0.0298 (12)	0.0462 (15)	0.0581 (16)	−0.0023 (10)	0.0036 (11)	−0.0075 (12)
C11	0.0359 (14)	0.0597 (17)	0.0589 (17)	−0.0007 (12)	0.0089 (12)	0.0180 (14)
C12	0.0344 (13)	0.082 (2)	0.0379 (14)	−0.0010 (13)	−0.0006 (11)	0.0131 (13)
C13	0.0474 (15)	0.0620 (18)	0.0423 (14)	−0.0006 (13)	0.0078 (12)	−0.0031 (13)
C14	0.0346 (12)	0.0452 (14)	0.0391 (13)	0.0069 (10)	0.0037 (10)	−0.0038 (11)
C15	0.0391 (13)	0.0524 (16)	0.0441 (14)	−0.0019 (11)	−0.0103 (11)	−0.0096 (12)
C16	0.0398 (13)	0.0411 (14)	0.0455 (14)	−0.0081 (11)	−0.0041 (11)	−0.0031 (11)
C17	0.0282 (11)	0.0429 (13)	0.0326 (12)	−0.0039 (10)	0.0034 (9)	−0.0037 (10)
C18	0.0366 (13)	0.0478 (15)	0.0402 (13)	−0.0089 (11)	−0.0007 (10)	−0.0085 (11)
C19	0.0419 (13)	0.0362 (13)	0.0458 (14)	−0.0024 (10)	0.0060 (11)	−0.0025 (11)
C20	0.0337 (12)	0.0430 (14)	0.0355 (12)	−0.0081 (10)	0.0015 (10)	−0.0027 (10)
C26	0.0471 (15)	0.0555 (17)	0.0517 (16)	0.0108 (13)	−0.0020 (12)	0.0031 (13)
O1	0.0293 (9)	0.0739 (13)	0.0442 (10)	0.0017 (8)	−0.0065 (7)	0.0121 (9)
N1	0.0266 (10)	0.0491 (12)	0.0393 (11)	0.0000 (8)	−0.0023 (8)	0.0093 (9)
O2	0.0403 (11)	0.0565 (12)	0.1079 (18)	−0.0095 (9)	−0.0029 (11)	−0.0127 (12)
O3	0.0340 (9)	0.0783 (13)	0.0472 (10)	−0.0111 (9)	0.0048 (8)	0.0139 (9)
Cl1	0.0836 (6)	0.0706 (5)	0.0591 (5)	0.0037 (4)	0.0180 (4)	0.0208 (4)
Cl2	0.0733 (5)	0.0911 (6)	0.0425 (4)	0.0114 (4)	−0.0080 (3)	0.0028 (4)
C21	0.034 (3)	0.038 (4)	0.037 (3)	0.001 (2)	0.008 (2)	−0.001 (2)
C22	0.029 (3)	0.048 (4)	0.054 (4)	−0.004 (3)	0.006 (2)	−0.001 (3)
C23	0.035 (3)	0.053 (4)	0.055 (3)	−0.002 (2)	−0.0049 (19)	−0.006 (3)
C24	0.040 (3)	0.067 (6)	0.039 (3)	0.001 (3)	−0.009 (2)	−0.003 (3)
C25	0.041 (3)	0.051 (4)	0.034 (3)	0.000 (3)	0.000 (2)	0.005 (3)
O4	0.0366 (16)	0.082 (2)	0.074 (2)	−0.0104 (14)	−0.0054 (13)	−0.0299 (17)
N2	0.029 (2)	0.041 (6)	0.031 (2)	−0.0078 (18)	0.0001 (16)	0.001 (3)
C21'	0.037 (5)	0.054 (9)	0.033 (5)	−0.003 (5)	−0.001 (4)	0.002 (5)
C22'	0.027 (6)	0.047 (6)	0.041 (5)	0.001 (4)	−0.001 (4)	0.005 (4)
C23'	0.032 (5)	0.050 (5)	0.046 (5)	0.005 (3)	−0.004 (3)	0.003 (3)
C24'	0.040 (5)	0.047 (7)	0.043 (5)	0.003 (4)	0.000 (4)	−0.001 (4)
C25'	0.034 (5)	0.037 (5)	0.035 (5)	0.002 (4)	0.009 (4)	0.001 (4)
O4'	0.035 (3)	0.046 (3)	0.075 (4)	0.004 (3)	0.003 (2)	0.000 (3)
N2'	0.038 (5)	0.027 (8)	0.036 (4)	−0.004 (3)	0.002 (3)	−0.002 (4)

### Geometric parameters (Å, º)

**Table:**

C1—C6	1.379 (3)	C18—H18	0.9300
C1—C2	1.380 (3)	C19—H19	0.9300
C1—C13	1.493 (3)	C20—O3	1.233 (3)
C2—C3	1.376 (3)	C20—N2	1.335 (7)
C2—H2	0.9300	C20—N2'	1.359 (14)
C3—C4	1.384 (3)	C26—Cl2	1.793 (3)
C3—H3	0.9300	C26—H26A	0.9700
C4—C5	1.380 (3)	C26—H26B	0.9700
C4—C7	1.495 (3)	O2—H2A	0.8200
C5—C6	1.373 (3)	C21—N2	1.489 (10)
C5—H5	0.9300	C21—C22	1.512 (8)
C6—H6	0.9300	C21—H21A	0.9700
C7—O1	1.235 (3)	C21—H21B	0.9700
C7—N1	1.337 (3)	C22—C23	1.511 (8)
C8—N1	1.461 (3)	C22—H22A	0.9700
C8—C9	1.508 (3)	C22—H22B	0.9700
C8—H8A	0.9700	C23—O4	1.391 (8)
C8—H8B	0.9700	C23—C24	1.521 (9)
C9—C10	1.510 (4)	C23—H23	0.9800
C9—H9A	0.9700	C24—C25	1.505 (8)
C9—H9B	0.9700	C24—H24A	0.9700
C10—O2	1.417 (3)	C24—H24B	0.9700
C10—C11	1.511 (4)	C25—N2	1.483 (9)
C10—H10	0.9800	C25—H25A	0.9700
C11—C12	1.514 (4)	C25—H25B	0.9700
C11—H11A	0.9700	O4—H4	0.8200
C11—H11B	0.9700	C21'—N2'	1.41 (2)
C12—N1	1.460 (3)	C21'—C22'	1.544 (16)
C12—H12A	0.9700	C21'—H21C	0.9700
C12—H12B	0.9700	C21'—H21D	0.9700
C13—Cl1	1.789 (3)	C22'—C23'	1.540 (14)
C13—H13A	0.9700	C22'—H22C	0.9700
C13—H13B	0.9700	C22'—H22D	0.9700
C14—C19	1.384 (3)	C23'—O4'	1.372 (14)
C14—C15	1.385 (4)	C23'—C24'	1.551 (15)
C14—C26	1.496 (3)	C23'—H23'	0.9800
C15—C16	1.373 (3)	C24'—C25'	1.538 (15)
C15—H15	0.9300	C24'—H24C	0.9700
C16—C17	1.383 (3)	C24'—H24D	0.9700
C16—H16	0.9300	C25'—N2'	1.411 (19)
C17—C18	1.380 (3)	C25'—H25C	0.9700
C17—C20	1.496 (3)	C25'—H25D	0.9700
C18—C19	1.380 (3)	O4'—H4'	0.8200
			
C6—C1—C2	118.6 (2)	N2'—C20—C17	119.6 (8)
C6—C1—C13	120.9 (2)	C14—C26—Cl2	110.88 (18)
C2—C1—C13	120.5 (2)	C14—C26—H26A	109.5
C3—C2—C1	120.8 (2)	Cl2—C26—H26A	109.5
C3—C2—H2	119.6	C14—C26—H26B	109.5
C1—C2—H2	119.6	Cl2—C26—H26B	109.5
C2—C3—C4	120.3 (2)	H26A—C26—H26B	108.1
C2—C3—H3	119.9	C7—N1—C12	120.33 (19)
C4—C3—H3	119.9	C7—N1—C8	125.23 (19)
C5—C4—C3	118.9 (2)	C12—N1—C8	113.28 (19)
C5—C4—C7	119.0 (2)	C10—O2—H2A	109.5
C3—C4—C7	121.8 (2)	N2—C21—C22	108.0 (10)
C6—C5—C4	120.5 (2)	N2—C21—H21A	110.1
C6—C5—H5	119.8	C22—C21—H21A	110.1
C4—C5—H5	119.8	N2—C21—H21B	110.1
C5—C6—C1	120.9 (2)	C22—C21—H21B	110.1
C5—C6—H6	119.5	H21A—C21—H21B	108.4
C1—C6—H6	119.5	C23—C22—C21	113.4 (10)
O1—C7—N1	121.9 (2)	C23—C22—H22A	108.9
O1—C7—C4	119.1 (2)	C21—C22—H22A	108.9
N1—C7—C4	118.95 (19)	C23—C22—H22B	108.9
N1—C8—C9	110.5 (2)	C21—C22—H22B	108.9
N1—C8—H8A	109.6	H22A—C22—H22B	107.7
C9—C8—H8A	109.6	O4—C23—C22	110.9 (6)
N1—C8—H8B	109.6	O4—C23—C24	106.8 (7)
C9—C8—H8B	109.6	C22—C23—C24	106.4 (10)
H8A—C8—H8B	108.1	O4—C23—H23	110.9
C8—C9—C10	111.0 (2)	C22—C23—H23	110.9
C8—C9—H9A	109.4	C24—C23—H23	110.9
C10—C9—H9A	109.4	C25—C24—C23	109.3 (10)
C8—C9—H9B	109.4	C25—C24—H24A	109.8
C10—C9—H9B	109.4	C23—C24—H24A	109.8
H9A—C9—H9B	108.0	C25—C24—H24B	109.8
O2—C10—C11	108.6 (2)	C23—C24—H24B	109.8
O2—C10—C9	112.8 (2)	H24A—C24—H24B	108.3
C11—C10—C9	109.2 (2)	N2—C25—C24	107.6 (10)
O2—C10—H10	108.7	N2—C25—H25A	110.2
C11—C10—H10	108.7	C24—C25—H25A	110.2
C9—C10—H10	108.7	N2—C25—H25B	110.2
C10—C11—C12	111.0 (2)	C24—C25—H25B	110.2
C10—C11—H11A	109.4	H25A—C25—H25B	108.5
C12—C11—H11A	109.4	C23—O4—H4	109.5
C10—C11—H11B	109.4	C20—N2—C25	119.2 (7)
C12—C11—H11B	109.4	C20—N2—C21	122.5 (8)
H11A—C11—H11B	108.0	C25—N2—C21	116.6 (9)
N1—C12—C11	110.3 (2)	N2'—C21'—C22'	115 (2)
N1—C12—H12A	109.6	N2'—C21'—H21C	108.5
C11—C12—H12A	109.6	C22'—C21'—H21C	108.5
N1—C12—H12B	109.6	N2'—C21'—H21D	108.5
C11—C12—H12B	109.6	C22'—C21'—H21D	108.5
H12A—C12—H12B	108.1	H21C—C21'—H21D	107.5
C1—C13—Cl1	111.73 (18)	C23'—C22'—C21'	106 (2)
C1—C13—H13A	109.3	C23'—C22'—H22C	110.6
Cl1—C13—H13A	109.3	C21'—C22'—H22C	110.6
C1—C13—H13B	109.3	C23'—C22'—H22D	110.6
Cl1—C13—H13B	109.3	C21'—C22'—H22D	110.6
H13A—C13—H13B	107.9	H22C—C22'—H22D	108.7
C19—C14—C15	118.6 (2)	O4'—C23'—C22'	115.4 (15)
C19—C14—C26	120.4 (2)	O4'—C23'—C24'	100.2 (15)
C15—C14—C26	121.1 (2)	C22'—C23'—C24'	113 (2)
C16—C15—C14	120.8 (2)	O4'—C23'—H23'	109.4
C16—C15—H15	119.6	C22'—C23'—H23'	109.4
C14—C15—H15	119.6	C24'—C23'—H23'	109.4
C15—C16—C17	120.6 (2)	C25'—C24'—C23'	113 (2)
C15—C16—H16	119.7	C25'—C24'—H24C	109.0
C17—C16—H16	119.7	C23'—C24'—H24C	109.0
C18—C17—C16	119.0 (2)	C25'—C24'—H24D	109.0
C18—C17—C20	121.3 (2)	C23'—C24'—H24D	109.0
C16—C17—C20	119.4 (2)	H24C—C24'—H24D	107.8
C19—C18—C17	120.4 (2)	N2'—C25'—C24'	115 (2)
C19—C18—H18	119.8	N2'—C25'—H25C	108.5
C17—C18—H18	119.8	C24'—C25'—H25C	108.5
C18—C19—C14	120.7 (2)	N2'—C25'—H25D	108.5
C18—C19—H19	119.7	C24'—C25'—H25D	108.5
C14—C19—H19	119.7	H25C—C25'—H25D	107.5
O3—C20—N2	122.4 (4)	C23'—O4'—H4'	109.5
O3—C20—N2'	121.2 (8)	C20—N2'—C21'	129.3 (18)
O3—C20—C17	118.9 (2)	C20—N2'—C25'	122.8 (15)
N2—C20—C17	118.2 (4)	C21'—N2'—C25'	107.6 (19)
			
C6—C1—C2—C3	1.0 (4)	C19—C14—C26—Cl2	101.6 (3)
C13—C1—C2—C3	179.7 (2)	C15—C14—C26—Cl2	−79.3 (3)
C1—C2—C3—C4	−2.1 (4)	O1—C7—N1—C12	−3.4 (4)
C2—C3—C4—C5	1.4 (4)	C4—C7—N1—C12	173.9 (2)
C2—C3—C4—C7	−172.8 (2)	O1—C7—N1—C8	163.5 (2)
C3—C4—C5—C6	0.3 (4)	C4—C7—N1—C8	−19.2 (4)
C7—C4—C5—C6	174.6 (2)	C11—C12—N1—C7	−135.0 (2)
C4—C5—C6—C1	−1.3 (4)	C11—C12—N1—C8	56.6 (3)
C2—C1—C6—C5	0.6 (4)	C9—C8—N1—C7	135.6 (3)
C13—C1—C6—C5	−178.0 (2)	C9—C8—N1—C12	−56.7 (3)
C5—C4—C7—O1	−52.0 (3)	N2—C21—C22—C23	52.6 (15)
C3—C4—C7—O1	122.2 (3)	C21—C22—C23—O4	−176.1 (9)
C5—C4—C7—N1	130.6 (2)	C21—C22—C23—C24	−60.4 (12)
C3—C4—C7—N1	−55.2 (3)	O4—C23—C24—C25	−177.4 (8)
N1—C8—C9—C10	56.2 (3)	C22—C23—C24—C25	64.1 (11)
C8—C9—C10—O2	−177.3 (2)	C23—C24—C25—N2	−61.6 (13)
C8—C9—C10—C11	−56.4 (3)	O3—C20—N2—C25	−0.8 (16)
O2—C10—C11—C12	179.8 (2)	C17—C20—N2—C25	171.0 (9)
C9—C10—C11—C12	56.4 (3)	O3—C20—N2—C21	163.4 (9)
C10—C11—C12—N1	−56.2 (3)	C17—C20—N2—C21	−24.7 (17)
C6—C1—C13—Cl1	−78.8 (3)	C24—C25—N2—C20	−138.2 (12)
C2—C1—C13—Cl1	102.6 (3)	C24—C25—N2—C21	56.6 (15)
C19—C14—C15—C16	−0.1 (4)	C22—C21—N2—C20	144.2 (14)
C26—C14—C15—C16	−179.2 (2)	C22—C21—N2—C25	−51.2 (16)
C14—C15—C16—C17	−1.3 (4)	N2'—C21'—C22'—C23'	63 (3)
C15—C16—C17—C18	1.3 (4)	C21'—C22'—C23'—O4'	67 (2)
C15—C16—C17—C20	174.8 (2)	C21'—C22'—C23'—C24'	−47 (2)
C16—C17—C18—C19	0.1 (4)	O4'—C23'—C24'—C25'	−82 (2)
C20—C17—C18—C19	−173.3 (2)	C22'—C23'—C24'—C25'	42 (2)
C17—C18—C19—C14	−1.5 (4)	C23'—C24'—C25'—N2'	−46 (3)
C15—C14—C19—C18	1.5 (4)	O3—C20—N2'—C21'	173 (2)
C26—C14—C19—C18	−179.4 (2)	C17—C20—N2'—C21'	−1 (4)
C18—C17—C20—O3	118.4 (3)	O3—C20—N2'—C25'	−15 (4)
C16—C17—C20—O3	−55.0 (3)	C17—C20—N2'—C25'	172 (2)
C18—C17—C20—N2	−53.7 (8)	C22'—C21'—N2'—C20	106 (3)
C16—C17—C20—N2	132.9 (8)	C22'—C21'—N2'—C25'	−68 (3)
C18—C17—C20—N2'	−67.9 (17)	C24'—C25'—N2'—C20	−117 (3)
C16—C17—C20—N2'	118.7 (17)	C24'—C25'—N2'—C21'	57 (3)

### Hydrogen-bond geometry (Å, º)

**Table:**

*D*—H···*A*	*D*—H	H···*A*	*D*···*A*	*D*—H···*A*
O2—H2*A*···O1^i^	0.82	2.18	2.789 (2)	132
O4—H4···O3^ii^	0.82	2.13	2.793 (3)	138
C11—H11*B*···O3^iii^	0.97	2.60	3.522 (3)	160
C26—H26*A*···O2^iv^	0.97	2.59	3.494 (4)	154

Symmetry codes: (i) *x*−1, *y*, *z*; (ii) *x*+1, *y*, *z*; (iii) −*x*+1, −*y*, −*z*+1; (iv) *x*, *y*+1, *z*.
