# Supplementary figures and images for: Crystal structure of [4-(chloro­meth­yl)phen­yl](4-hy­droxy­piperidin-1-yl)methanone
Source: Acta Crystallogr E Crystallogr Commun. 2015 Sep 12;71(Pt 10):o703–4. doi: 10.1107/S2056989015016096 (PMC4647401; doi:10.1107/S2056989015016096)

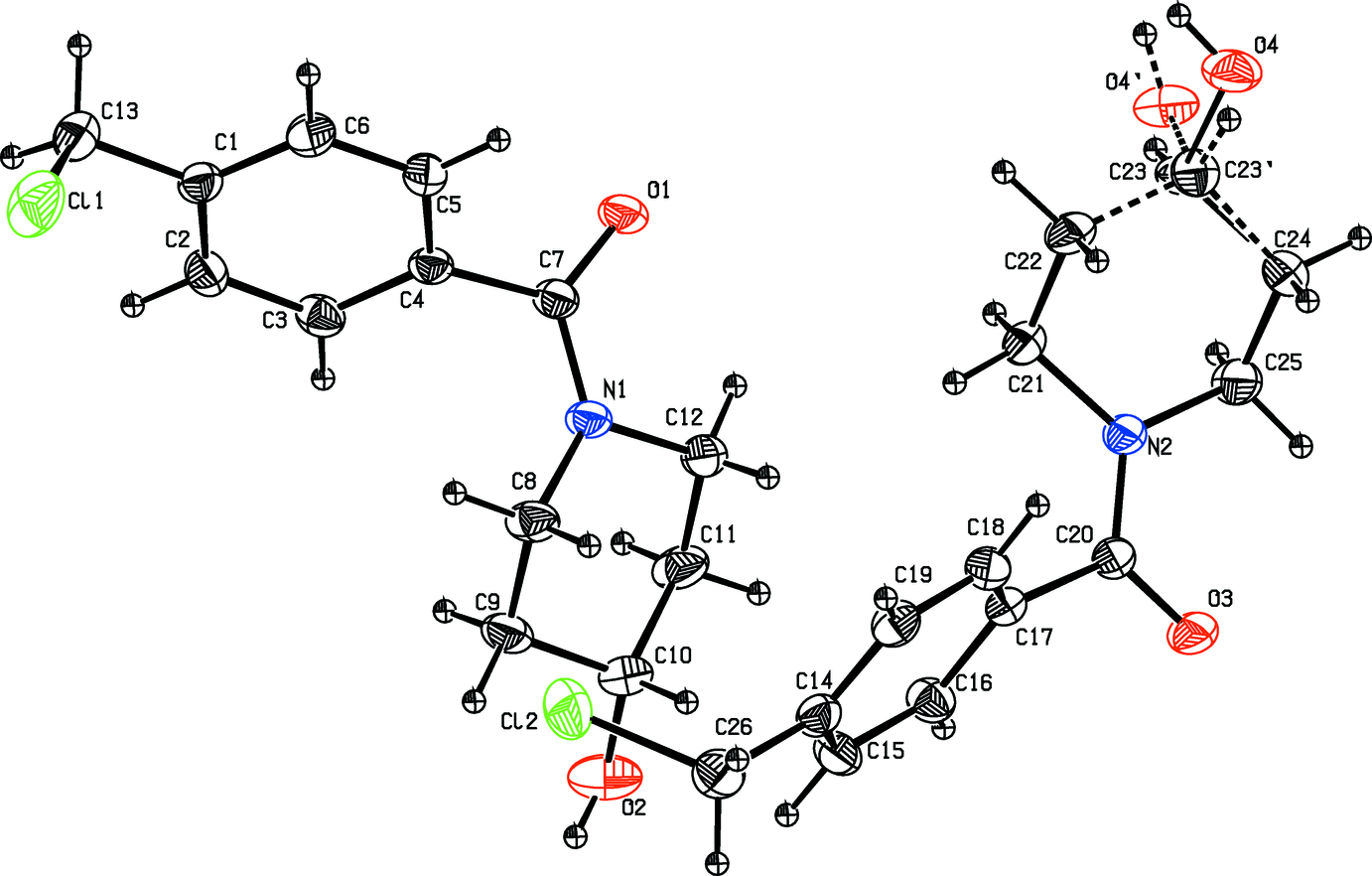

Supplement: Supplementary file 4 [file e-71-0o703-fig1.tif]

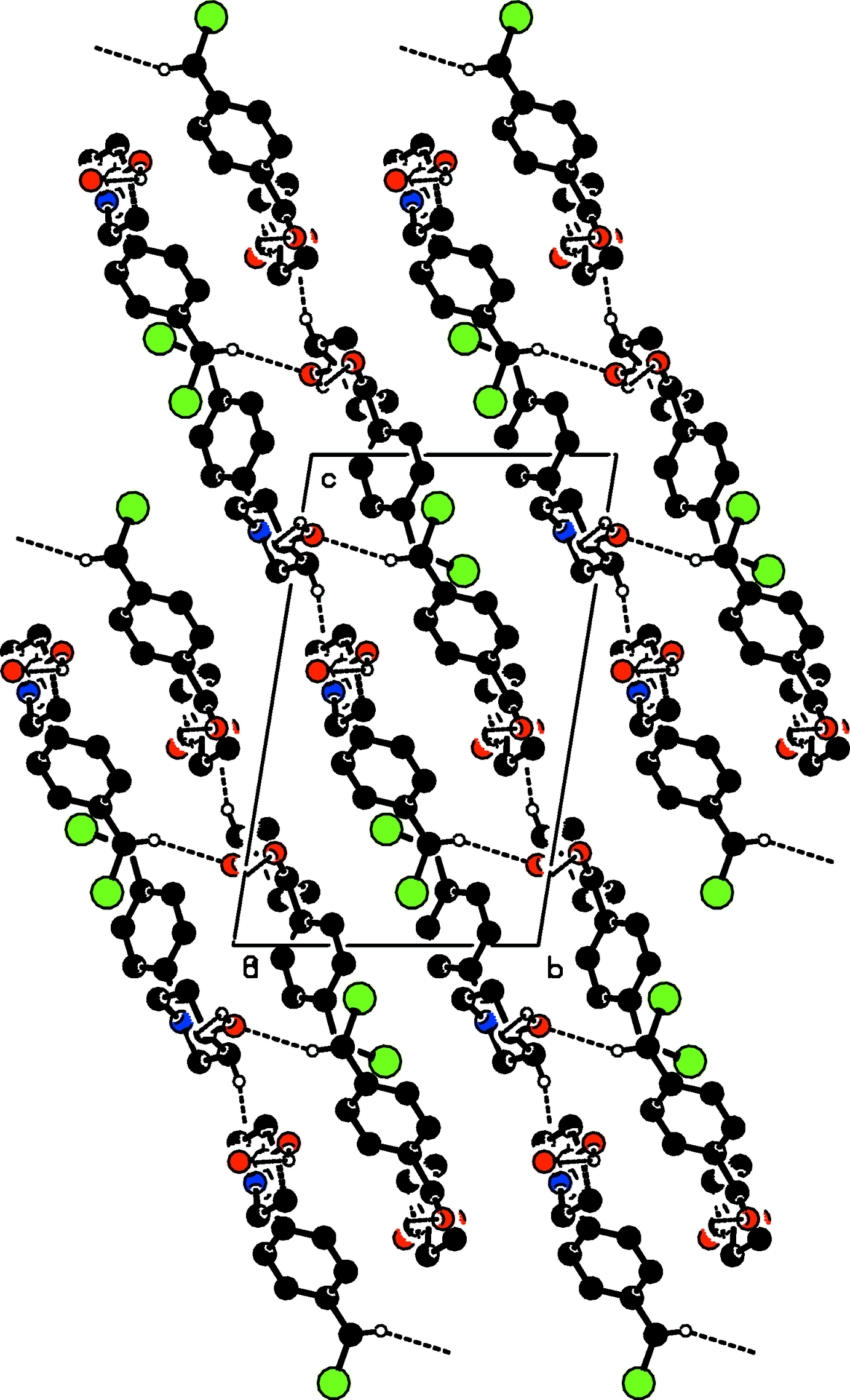

Supplement: Supplementary file 5 [file e-71-0o703-fig2.tif]
